# A 6-month “self-monitoring” lifestyle modification with increased sunlight exposure modestly improves vitamin D status, lipid profile and glycemic status in overweight and obese Saudi adults with varying glycemic levels

**DOI:** 10.1186/1476-511X-13-87

**Published:** 2014-05-26

**Authors:** Nasser M Al-Daghri, Hanan Alfawaz, Naji J Aljohani, Yousef Al-Saleh, Kaiser Wani, Abdullah M Alnaami, Mohammad Alharbi, Sudhesh Kumar

**Affiliations:** 1 Prince Mutaib Chair for Biomarkers of Osteoporosis, Biochemistry Department, King Saud University, PO Box, 2455, Riyadh 11451, Saudi Arabia; 2 Biomarkers Research Program, Biochemistry Department, College of Science, King Saud University, Riyadh, Saudi Arabia; 3 Department of Food Science and Nutrition, College of Food Science and Agriculture King Saud University, Riyadh, Saudi Arabia; 4 Specialized Diabetes and Endocrine Center, King Fahad Medical City, Faculty of Medicine, King Saud bin Abdulaziz University for Health Sciences, Riyadh 11525, Saudi Arabia; 5 College of Medicine, King Saud bin Abdulaziz University for Health Sciences, Riyadh 11461, Saudi Arabia; 6 Diabetes Centers and Units Administration, Ministry of Health, Riyadh, Saudi Arabia; 7 Division of Metabolic and Vascular Health, Clinical Sciences Research Institute, University Hospitals Coventry and Warwickshire Trust, Walsgrave, Coventry CV2 2DX, UK

**Keywords:** Vitamin D, Self-monitoring, Diabetes mellitus, Pre-diabetes, Obesity

## Abstract

**Background:**

The over-all age-adjusted prevalence of diabetes mellitus type 2 (DMT2) in Saudi Arabia is unprecedented at 31%. Aggressive measures should be done to curb down increasing incidence. In this prospective 6-month study we aim to determine whether a self-monitoring, life-style modification program that includes increased sunlight exposure confer improvement in vitamin D status and health benefits among adult Saudi overweight and obese patients with varying glycemic status.

**Methods:**

A total of 150 overweight and obese Saudi adults with varying glycemic status aged 30–60 years were included in this study. They were divided into 3 groups (Non-DMT2, Pre-diabetes and DMT2). Baseline anthropometrics and blood glucose were taken at baseline and after 6 months. Fasting blood sugar, lipid profile, calcium, albumin and phosphate were measured routinely. Serum 25(OH) vitamin D was measured using standard assays. Within the time period they were instructed to reduce total intake of fat, increased fiber intake and increase sun exposure.

**Results:**

In all groups there was a significant improvement in vitamin D levels as well as serum triglycerides, LDL- and total cholesterol. However, a significant increase in serum glucose levels was noted in the non-DMT2 group, and a significant decrease in HDL-cholesterol in both non-DMT2 and pre-diabetes group. In the pre-diabetes group, 53.2% were able to normalize their fasting blood levels after 6 months, with 8.5% reaching the DMT2 stage and 38.3% remaining pre-diabetic. In all groups there was a significant increase in the prevalence of hypertension.

**Conclusion:**

Improving vitamin D status with modest lifestyle modifications over a short-period translates to improvement in lipid profile except HDL-cholesterol among overweight and obese Saudi adults, but not BMI and blood pressure. Findings of the present study merit further investigation as to whether full vitamin D status correction can delay or prevent onset of DMT2.

## Background

If the prevalence of diabetes mellitus type 2 (DMT2) continues to increase at the current rate, the global burden of this disease will swell between from 171 million in 2000, to 366 million patients in 2030 [1]. Furthermore, healthcare expenditures on DMT2 alone will skyrocket from US$376 billion in 2010 to US$490 billion in 2030 [2]. The Middle East region has not been spared from this scourge and currently is among those worst-hit [1]. This global epidemic, shared by both industrialized and developing countries, has stimulated increased public awareness of the disease, the identification of risk factors and the knowledge that DMT2 can be delayed and, even better, prevented [3-5]. Recognition of the importance of glycemic control in the prevention of the complications and morbidity of DMT2 has led to worldwide campaigns for modifications in lifestyle and an intensive search for better anti-diabetes medications [6-8].

Harboring pre-diabetes, or state of intermediate hyperglycemia (plasma glucose levels higher than normal, but lower than the threshold of DM), puts an individual at high risk not only for developing DM but associated complications as well. Among the Gulf neighboring countries, the prevalence of pre-diabetes based on several low-moderate sample-sized cross-sectional studies is moderately high: 9.0% in Saudi Arabia [9], 13.8% in Qatar [10], 40.9% in Oman [11], and 24.2% in United Arab Emirates (UAE) [12]. According to an expert from the American Diabetes Association (ADA), almost 70% of the pre-diabetes population will eventually develop DMT2, and in over a 3- to 5-year period, persons who harbor pre-diabetes have a 5- to 15-fold higher risk of further progressing to DMT2 as opposed to their normoglycemic counterparts [13,14]. Having mentioned such and considering the global economic burden of DMT2, not to mention the perpetual obstacles to deliver high-quality standard care to DMT2 patients, the challenge to fight DMT2 through prevention is a compelling case worthy of attention and enormous investigation.

Fortunately during the recent years, there has been accumulating evidence from various interventional studies on the beneficial effects of improving vitamin D status among patients with DMT2 and the full metabolic syndrome [15-17]. This suggests some important clinical implications among patients at risk of developing DMT2. However, there is limited information on the role of improving vitamin D status among patients with pre-diabetes, specifically in this region where the prevalence of both DMT2 and vitamin D deficiency are high. Having mentioned such, the present interventional study aims to determine whether a self-monitoring, life-style modification program that includes increased sunlight exposure confer improvement in vitamin D status and health benefits among adult Saudi overweight and obese patients with varying glycemic status.

## Methods

### Site and duration of the study

This is a multi-center, interventional study to be conducted at the primary care centers of the RIYADH Cohort, a capital-based screening program involving more than 17000 Saudi subjects randomly selected to participate in epidemiologic studies conducted by the Biomarkers Research Program (BRP) of King Saud University (KSU) and the Ministry of Health (MOH), Riyadh, Saudi Arabia.

### Subjects

A total of 200 Saudi adult males and females aged 30–60 years old with unknown DM status and varying body mass indices (BMI) were initially recruited to take part in this 6-month interventional study. These subjects were recruited from the community participants of the Saudi Diabetes Charity Foundation based in Riyadh, Saudi Arabia. Out of the 200 subjects, 50 dropped out for various reasons (non-compliance, change of medication, lost to follow up, etc.). They were divided into 3 groups based on baseline fasting blood glucose (FBG) levels: Non-DMT2 group (FBG < 5.6 mmol/l), Pre-DMT2 group (FBG 5.6-6.9 mmol/l) and DMT2 group (FBG ≥ 7.0 mmol/l). Non-consenting subjects, pregnant subjects, and anyone with complications such as renal, neurologic, hepatic and pulmonary diseases were excluded as well as acute conditions that require immediate medical attention. The study was performed in accordance with the the ethical principles in the Declaration of Helsinki as well as with the ICH Note for Guidance on Good Clinical Practice. Approval from the ethics committee of the College of Science, KSU was sought prior to study commencement.

### Clinical assessment

Baseline anthropometric measurements were noted by the assigned and registered primary care physician and nurse. These included height (cm) and weight (kg) from which BMI was calculated [weight (kg)/height (m^2^)]. Blood pressure (mmHg) was taken twice after 30 minutes rest using the conventional mercurial sphygmomanometer and the average was recorded. All measurements were repeated after 6 months.

### Laboratory parameters

All subjects were requested to submit an over-night fasting blood samples from which the different metabolic parameters were assessed. Serum FBG, complete lipid profile, serum albumin, calcium and phosphate were determined using routine laboratory methods. Serum 25(OH) vitamin D was measured using an enzyme-linked immunosorbent assay (ELISA) (IDS Ltd., Boldon Colliery, Tyne & Wear, UK). The inter- and intra-assay variabilities were 5.3% and 4.6%. All measurements were done in a DEQAS- (Vitamin D External Quality Assessment Scheme) participating laboratory, the Biomarkers Research Program (BRP) of King Saud University, Riyadh, KSA.

### Lifestyle intervention

All subjects were oriented and educated about lifestyle modifications from successful intervention programs done elsewhere [18,19], advised and followed-up regularly during specified visits. In brief, the intervention program has specific goals which include weight reduction of 5% or more, total intake of fat to < 30% of energy consumed, intake of saturated fat < 10% of energy consumed, increased fiber intake to at least 15 g/1000 kcal and moderate exercise for at least 30 minutes per day (30 minute walk, 5 times a week). Some of these goals are exemplified as taken from the Diabetes Prevention Program (DPP): eat smaller portions; drink more water and little or no sugar-containing beverages each day; choose leaner cuts of beef, eat white meat of turkey and chicken more often, eat fish high in omega-3; fatty acids, and no more than 340 gram per week; increase intake of whole fruits and vegetables; choose whole grains like rolled oats, barley, bran, and 100% whole grain bread instead of refined, processed carbohydrates like baked products made with white flour; choose low or no fat dairy products; use unsaturated vegetable oils that are liquid at room temperature like olive, canola, peanut, safflower, sunflower, corn, soybean and cottonseed oils, and use soft-tub, squeeze or spray margarine; eat at regular mealtimes; use low-fat food preparation (grilling, broiling, boiling, steaming, etc.…); eat breakfast and reduce frequency of eating out, especially in fast food restaurants [20].

The orientation and intervention were conducted by a certified nutritionist, physician, nurse and physical therapists, who were also the same team that reinforced instructions to the subjects on a regular basis. In addition, they were advised to increase sunlight exposure during the entire 6 months of the study. In all subjects, “self-monitoring” was introduced, and they were subjected to regular calls from the investigator to keep track of the progress.

### Data analysis

Collected data was analyzed using SPSS version 16.5 for windows (Chicago, Illinois, USA). Frequencies were presented in percent (%) cases and Chi-square was used to determine differences. Paired T-test was done to determine intervention effects in anthropometric and metabolic parameters at baseline and after 6 months. Bivariate correlation coefficients between the variables were determined. *P*-value ≤ 0.05 will be considered significant.

## Results

Table 1 highlights the characteristics of all subjects and the non-diabetic group. In all subjects, no significant changes were observed in BMI, blood pressure and fasting blood glucose overtime. Serum triglycerides, total cholesterol, LDL- and HDL-cholesterol significantly decreased after 6 months compared to baseline (*p*-values 0.048, 5.3 x 10^−6^, 2.5 x 10^−5^ and 3.0 x 10^−4^, respectively). On the other hand, baseline serum calcium and albumin were noted to be significantly higher as compared to 6 months (*p*-values 1.1 x 10^−7^ and 0.026, respectively. There was also a significant increase in levels of circulating 25(OH) vitamin D from baseline to 6 months (*p*-value 3.3 x 10^−4^). In the non-diabetic group, baseline BMI and blood pressure remained almost unchanged after 6 months follow-up. However, a significant increase in fasting serum glucose was noted after 6 months (*p*-value 0.0002). With regards to lipids, changes in baseline serum triglycerides, total and LDL-cholesterol were not significant after 6 months follow-up. However, follow-up HDL-cholesterol was significantly higher as compared to baseline levels (*p*-value 0.0002). Baseline serum albumin levels significantly increased after 6 months as well as baseline serum 25(OH) vitamin D (*p*-values 0.004, 0.004 respectively).

**Table 1:** Anthropometric and metabolic changes of all subjects and non-DM group over time

	**All subjects**			**Non-DMT2 group**		
	**Baseline**	**6 months**	**P-value**	**Baseline**	**6 months**	**P-value**
M/F	37/113			15/51		
Age (years)	44.78 ± 0.93			39.8 ± 1.44		
BMI (kg/m^2^)	32.3 ± 0.5	32.2 ± 0.5	NS	32.6 ± 0.8	32.5 ± 0.8	NS
Systolic blood pressure (mmHg)	115.7 ± 1.7	115.4 ± 1.8	NS	115.4 ± 2.4	114.6 ± 2.5	NS
Diastolic blood pressure (mmHg)	84.8 ± 2.2	85.1 ± 24.7	NS	81.2 ± 3.3	82.6 ± 3.3	NS
Glucose (mmol/l)	6.1 ± 0.2	6.0 ± 0.2	NS	4.6 ± 0.1	5.2 ± 0.1	0.0002
Triglycerides (mmol/l)	1.7 ± 0.07	1.6 ± 0.06	0.048	1.62 ± 0.1	1.47 ± 0.08	NS
Total cholesterol (mmol/l)	4.7 ± 0.09	4.3 ± 0.08	5.3 × 10^−6^	4.6 ± 0.13	4.3 ± 0.11	NS
HDL-cholesterol (mmol/l)	1.1 ± 0.03	0.87 ± 0.05	3.0 × 10^−4^	1.1 ± 0.04	0.84 ± 0.07	0.002
LDL-cholesterol (mmol/l)	3.5 ± 0.11	3.1 ± 0.09	2.5 × 10^−5^	3.3 ± 0.14	3.06 ± 0.12	NS
Calcium (mmol/l)	2.1 ± 0.01	2.2 ± 0.01	1.1 × 10^−7^	2.07 ± 0.02	2.23 ± 0.02	0.0008
Corrected calcium (mmol/l)	2.2 ± 0.01	2.3 ± 0.01	7.3 × 10^−8^	2.17 ± 0.02	2.28 ± 0.02	0.001
Phosphate (mmol/l)	1.08 ± 0.05	1.11 ± 0.02	NS	1.08 ± 0.12	1.09 ± 0.02	NS
Albumin (g/l)	36.4 ± 0.46	37.6 ± 0.37	0.026	35.2 ± 0.68	37.6 ± 0.6	0.004
25-OH D (nmol/l)	45.7 ± 2.2	55.8 ± 2.7	3.3 x 10^−4^	42.3 ± 2.8	53.6 ± 3.6	0.004

**Note**: Data presented as mean ± standard error; NS – not significant; significance at *p* < 0.05.

Table 2 shows the metabolic changes in both the DMT2 group and the pre-diabetes group. In both groups, no significant improvements were observed in BMI, blood pressure, serum glucose and triglycerides over time. However, and also in both groups, a significant decrease was observed in serum total and LDL-cholesterol (DMT2 group p-values 0.0004 and 7.6 x 10^−5^; pre-DM group *p*-values 0.002 and 0.017, respectively. Changes in HDL-cholesterol were noted only in the pre-DM group, in which baseline levels were significantly higher than after 6 months (*p*-value 0.009). Serum calcium levels on the other hand significantly increased from baseline to 6 months in both groups (DMT2 group *p*-value 0.015; pre-DM group = 0.012). Lastly, improvements in 25(OH) vitamin D levels were noted only in the pre-DM group, with borderline significance (*p*-value 0.05).

**Table 2:** Anthropometric and metabolic changes of DM and pre-diabetes group over time

	**DMT2 group**			**Pre-diabetes group**		
	**Baseline**	**6 months**	**P-value**	**Baseline**	**6 months**	**P-value**
M/F	8/29			14/33		
Age (years)	47.69 ± 1.45			48.85 ± 1.46		
BMI (kg/m^2^)	31.8 ± 1.0	32.1 ± 0.9	NS	32.2 ± 0.97	32.0 ± 0.92	NS
Systolic blood pressure (mmHg)	115.7 ± 3.6	116.2 ± 4.4	NS	116.3 ± 3.5	115.8 ± 3.2	NS
Diastolic blood pressure (mmHg)	90.8 ± 4.9	89.7 ± 5.6	NS	85.3 ± 3.5	85.0 ± 3.8	NS
Glucose (mmol/l)	8.6 ± 0.4	7.8 ± 0.4	NS	6.1 ± 0.05	5.8 ± 0.17	NS
Triglycerides (mmol/l)	2.0 ± 0.17	1.9 ± 0.12	NS	1.7 ± 0.12	1.6 ± 0.11	NS
Total cholesterol (mmol/l)	5.0 ± 0.21	4.3 ± 0.17	0.0004	4.8 ± 0.14	4.3 ± 0.13	0.002
HDL-cholesterol (mmol/l)	0.98 ± 0.06	0.94 ± 0.11	NS	1.07 ± 0.05	0.86 ± 0.08	0.009
LDL-cholesterol (mmol/l)	3.8 ± 0.3	2.97 ± 0.2	7.6 x 10^−5^	3.6 ± 0.18	3.1 ± 0.16	0.017
Calcium (mmol/l)	2.1 ± 0.03	2.2 ± 0.04	0.015	2.09 ± 0.02	2.18 ± 0.02	0.012
Corrected calcium (mmol/l)	2.2 ± 0.02	2.3 ± 0.03	0.002	2.15 ± 0.02	2.23 ± 0.02	0.005
Phosphate (mmol/l)	1.08 ± 0.03	1.1 ± 0.04	NS	1.08 ± 0.02	1.08 ± 0.02	NS
Albumin (g/l)	37.4 ± 0.9	38.1 ± 0.7	NS	37.4 ± 0.87	37.3 ± 0.52	NS
25-OH D (nmol/l)	46.8 ± 3.6	54.7 ± 4.8	NS	49.3 ± 5.2	60.7 ± 5.8	0.05

**Note**: Data presented as mean ± standard error; NS – not significant; significance at *p* < 0.05.

Table 3 shows the associations of serum 25(OH) vitamin D to baseline anthropometric and metabolic parameters as well as after 6 months. In all subjects, only serum calcium was positively associated with 25(OH) vitamin D levels (R = 0.19; *p*-value < 0.05). This was also true for the pre-DM group (R = 0.27; *p*-value 0.05) while for the non-DMT2 group, serum calcium was significantly associated with 25(OH) vitamin D only at baseline) R = 0.27; *p*-value < 0.05). With regards to lipids, serum triglycerides and total cholesterol were both negatively and significantly associated with 25(OH) vitamin D levels, but only in the pre-DM group at baseline (R = −0.27 and −0.29, respectively). In the DMT2 group, only serum phosphate was significantly associated with 25(OH) vitamin D levels (R = 0.34; *p*-value < 0.05), and this was after 6 months. The rest of the associations were non-contributory.

**Table 3:** Associations of 25(OH) vitamin D to metabolic parameters according to groups

	**ALL subjects N = 150**		**Non-DMT2 group N = 67**		**Pre-DM group N = 47**		**DMT2 group N = 37**	
	**Baseline**	**6 months**	**Baseline**	**6 months**	**Baseline**	**6 months**	**Baseline**	**6 months**
BMI (kg/m^2^)	−0.10	−0.11	0.06	0.07	−0.19	−0.34*	−0.06	−0.03
Systolic blood pressure (mmHg)	0.10	−0.04	0.05	0.00	0.01	−0.02	0.21	−0.20
Diastolic blood pressure (mmHg)	0.03	−0.05	0.08	−0.10	−0.06	−0.18	−0.04	0.16
Glucose (mmol/l)	0.07	0.15	0.05	0.04	0.008	0.23	0.02	0.20
Triglycerides (mmol/l)	−0.04	−0.04	0.04	0.04	−0.11	−0.27*	−0.14	0.18
Total cholesterol (mmol/l)	−0.10	−0.15	−0.02	−0.10	−0.20	−0.29*	−0.11	−0.03
HDL-cholesterol (mmol/l)	−0.06	−0.05	0.05	−0.13	−0.28	−0.02	0.09	0.12
LDL-cholesterol (mmol/l)	−0.05	−0.14	0.02	−0.06	−0.05	−0.22	−0.15	−0.14
Calcium (mmol/l)	0.05	0.19*	0.27*	0.23	−0.04	0.27*	−0.32	0.10
Corrected calcium (mmol/l)	−0.02	0.15	0.20	0.17	−0.23	0.22	−0.11	0.09
Phosphate (mmol/l)	−0.02	0.07	0.19	0.16	−0.23	−0.19	0.00	0.34*
Albumin (g/l)	0.07	0.09	0.12	0.12	0.13	0.06	−0.19	0.08

Note: Data presented as coefficient (R); *denotes significance at *p* < 0.05.

**Figure 1:**
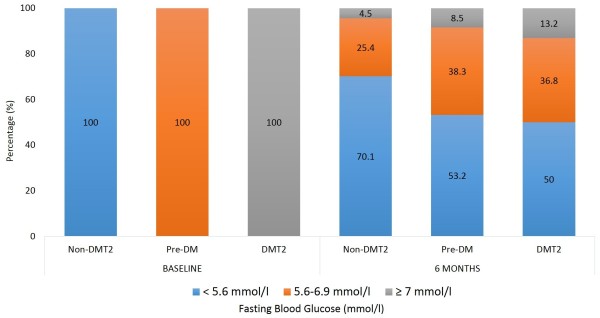
Prevalence of fasting glycemic states at baseline and after 6 months in non-DMT2, Pre-DM and DMT2 groups.

Lastly, Table 4 shows the changes in the cardiometabolic risk factors from baseline and after 6 months. In Figure 1, the most notable changes were observed in the glycemic status, in which all those who were non-DMT2 at baseline dropped to 70% after follow-up, with 25.4% under the category of pre-DM and 4.5% classified under DMT2. In the baseline pre-DM group, 53.2% were able to normalize their fasting blood levels after 6 months, with 8.5% reaching the DMT2 stage and 38.3% remaining pre-diabetic. Finally in the baseline DMT2 group, 13.2% were able to normalize their fasting blood glucose after 6 months, with 36.8% also improving but still in the pre-diabetes stage and 50% remaining well within the cut-off of DMT2. In all groups there was a significant increase in the prevalence of hypertension, while a significant decrease in the prevalence of hypertriglyceridemia was observed in the pre-DM group. Regardless of the glycemic status at baseline, there was a significant improvement in vitamin D status, with baseline vitamin D deficiency dropping significantly in all groups, but still high over-all (Table 4).

**Table 4:** Changes in risk factors over time

	**Non-DMT2 group N = 67**			**Pre-DM group N = 47**			**DMT2 group N = 37**		
	**Baseline**	**6 months**	**P-value**	**Baseline**	**6 months**	**P-value**	**Baseline**	**6 months**	**P-value**
Obesity	64.7	64.7	NS	64.3	61.9	NS	64.7	67.6	NS
Hypertension	23.6	26.0	0.006	21.4	34.3	0.0001	33.3	51.6	0.008
Hypertriglyceridemia	32.4	32.4	NS	46.8	38.3	0.04	48.6	52.6	NS
Vitamin D deficiency	67.6	52.2	0.009	55.3	52.2	0.008	62.2	48.6	0.04

**Note**: Data presented as percentages (%); NS – not significant; significance at *p* < 0.05.

## Discussion

The significant finding of the present interventional study was that the over-all improvement in the vitamin D status of the cohort after 6 months, as evidenced by the significant decrease in mean vitamin D levels and vitamin D deficiency prevalence, led to modest improvements at most, in the lipid profile that includes triglycerides, LDL- and total cholesterol, with no improvements observed in BMI and blood pressure. Furthermore, vitamin D status was not associated with majority of the metabolic parameters measured, with only triglycerides showing a positive association among patients with pre-diabetes and serum calcium in all subjects which was expected, considering that vitamin D is the main regulator of calcium homeostasis. This lack of promising clinical benefits highlights several caveats in the intervention program, particularly the “self-monitoring” strategy that the investigators employed. Nevertheless, interventional studies done elsewhere among patients with pre-diabetes also observed that raising vitamin D levels either through supplementation for 3 months one year had no effect on insulin sensitivity, pathophysiology of pre-diabetes and development of diabetes [21,22].

Another possible explanation for the lack of profound clinical improvement was that despite the significant improvement of vitamin D status across all groups, the BMI status remained constant, which is the main target of successful programs designed to reduce diabetes risk. The biggest and most convincing documentation for the risk-reducing benefits of lifestyle modification in the USA were studies from the Diabetes Prevention Program (DPP), which apparently was also the first randomized trial to compare lifestyle and a pharmacologic intervention to placebo [20]. Based on the DPP plan, weight reduction was the most powerful predictor of reduced diabetes incidence with an over-all 16.0% risk reduction for every 1 kg of reduced weight [23]. Nevertheless, people who achieved physical exercise instead of weight loss also experienced significant reduction in diabetes risk by as much as 44%. Modifications in physical activity and reduction of calories from dietary fat predicted weight loss, and this weight loss translated to lower diabetes risk, further confirming the premise that intercessions to decrease diabetes risk should target weight reduction especially in the overweight and obese population. It is worthy to note however that despite the lack of significant BMI loss, the glycemic status of the DMT2 group improved, with 50% of the subjects having lower fasting blood glucose levels as compared to their baseline levels. This however is overshadowed by the control group, who, in sharp contrast, had significantly higher mean levels of serum glucose after the intervention.

Another finding worthy of mention is the significant decrease in HDL-cholesterol in all groups despite improvements in the rest of the lipid profile. This finding is in line with several recently published studies questioning whether vitamin D status correction, at least among those who are vitamin D deficient, have no meaningful clinical changes in lipid concentrations, if not negative effect among those who were not vitamin D deficient [24,25]. This may have been the case in the present cohort, where vitamin D sufficiency is present within 30-40% across groups. Low HDL-cholesterol is already the most common metabolic syndrome component among Saudis with 90% of adults harboring such condition [26], and if previous claims are true on the negative effects on lipids on those patients who are not vitamin D deficient, then further investigations are needed to target only those who are most at risk for vitamin D deficiency, and exclude those whose vitamin D status are normal. Emerging indices for insulin resistance such as visceral adiposity index (VAI) are also suggested to be included in future studies to probe specific improvements outside conventional parameters used in the present study [27].

The present study has several caveats and findings should be interpreted with caution. It may have been underpowered with a short duration of intervention. The method of “self-monitoring” have made the results less beneficial but still favorable, since compliance and adherence is highly linked to the success of the program. Season should have been taken into consideration since it affects vitamin D status in the region [28]. Nevertheless the study has merits in being the first interventional study to observe the effects of sub-optimal vitamin D status replenishment in overweight and obese adults with varying degrees of glycemia in the Middle East.

## Conclusion

In summary, self-monitoring lifestyle modification coupled with increased sun exposure for 6 months improved, but not corrected vitamin D status, with no immediate impact in BMI, blood pressure and glucose levels among Saudi overweight and obese patients with pre-diabetes and DMT2. Significant improvements were most notable in lipid profile, with the exception of HDL-cholesterol, which decreased in all groups. Further interventional studies with a cohort exclusively to those with vitamin D deficiency and a more aggressive approach such as full vitamin D correction through supplementation in the implementation of lifestyle modifications is warranted to elicit a more favorable clinical improvement.

## Competing interests

The authors have no conflict of interest to disclose related to this study.

## Authors’ contributions

NMA and NJA conceived the study, oversaw study execution, and contributed to manuscript writing. HA, MA and YA recruited subjects and collected data. KW and AMA recruited subjects and analyzed samples. NMA performed data analysis and AMA wrote the manuscript. SK reviewed/edited the final version of the manuscript. All authors approved the final manuscript.
